# Fatal multifocal *Pasteurella multocida* infection: a case report

**DOI:** 10.1186/s13104-015-1232-7

**Published:** 2015-07-02

**Authors:** Mathieu Guilbart, Elie Zogheib, Abdel Hakim Hchikat, Kahina Kirat, Linda Ferraz, Anne-Marie Guerin-Robardey, Faouzi Trojette, Mona Moubarak-Daher, Hervé Dupont

**Affiliations:** Department of Anesthesiology and Critical Care Medicine, Amiens University Medical Center, Amiens, France; INSERM UMR 1088, Jules Verne University of Picardy, Amiens, France; Department of Orthopedic Surgery, Amiens University Medical Center, Amiens, France; Department of Anesthesiology and Critical Care Medicine, Beauvais Medical Center, Beauvais, France; Département d’Anesthésie-Réanimation, CHU d’Amiens Picardie, 80054 Amiens cedex, France

**Keywords:** *Pasteurella* infection, Endocarditis, Soft tissue infection

## Abstract

**Background:**

In humans, *Pasteurella multocida* infections are usually limited to the soft tissues surrounding a lesion. However, *P. multocida* can also cause systemic infections (such as pneumonia, lung abscess, peritonitis, endocarditis, meningitis and sepsis)—especially in patients with other underlying medical conditions.

**Case presentation:**

We report on a case of fulminant *P. multocida* bacteremia at several sites (soft tissues, endocarditis and joints) on a white European man. Despite surgery and intensive medical care, the patient died.

**Conclusions:**

The present case emphasizes the importance of appropriate initial treatment of skin wounds. Patients at risk should be aware of the possible consequences of being bitten, scratched or licked by their pet.

**Electronic supplementary material:**

The online version of this article (doi:10.1186/s13104-015-1232-7) contains supplementary material, which is available to authorized users.

## Background

The facultative anaerobe *Pasteurella multocida* is a fermentative, Gram-negative coccobacillus often found in the upper respiratory tracts of healthy fowls and domesticated species (especially cats and dogs) [1]. It can cause infections in humans, usually as a result of being scratched, bitten or licked by cat or dog. In the present report, we describe a fatal case of *P. multocida*-induced endocarditis and joints in a white European patient with several comorbidities (Additional file 1).

## Case presentation

A 74-year-old man was admitted to hospital for septic shock. He had been suffering from fever, weakness, and erysipelas of the right leg for the previous month. He mentioned that his pet dog had licked a skin wound, and had received two courses of antibiotics (amoxicillin + clavulanic acid). The comorbidities including atrial fibrillation, hypertension, venous leg ulcers, obesity, alcoholism and aortic valve stenosis. The patient had undergone total right knee joint replacement 1 year previously.

On admission, the patient presented with fever (38.1°C), hypotension (80/50 mmHg) and a known systolic aortic murmur. He had an elevated leukocyte count (13 × 10^9^ L^−1^), high C-reactive protein (340 mg L^−1^, N value <5) and procalcitonin (11 μg L^−1^, N value <0.5) levels and mild renal impairment (creatinine clearance: 32 ml min^−1^, serum creatinine: 181 µmol L^−1^, serum urea: 21 mmol L^−1^). Urine and blood cultures were sent to the microbiology lab. Fluid resuscitation and empirical antibiotic treatment therapy (with piperacillin + tazobactam) were initiated.

**Figure 1:**
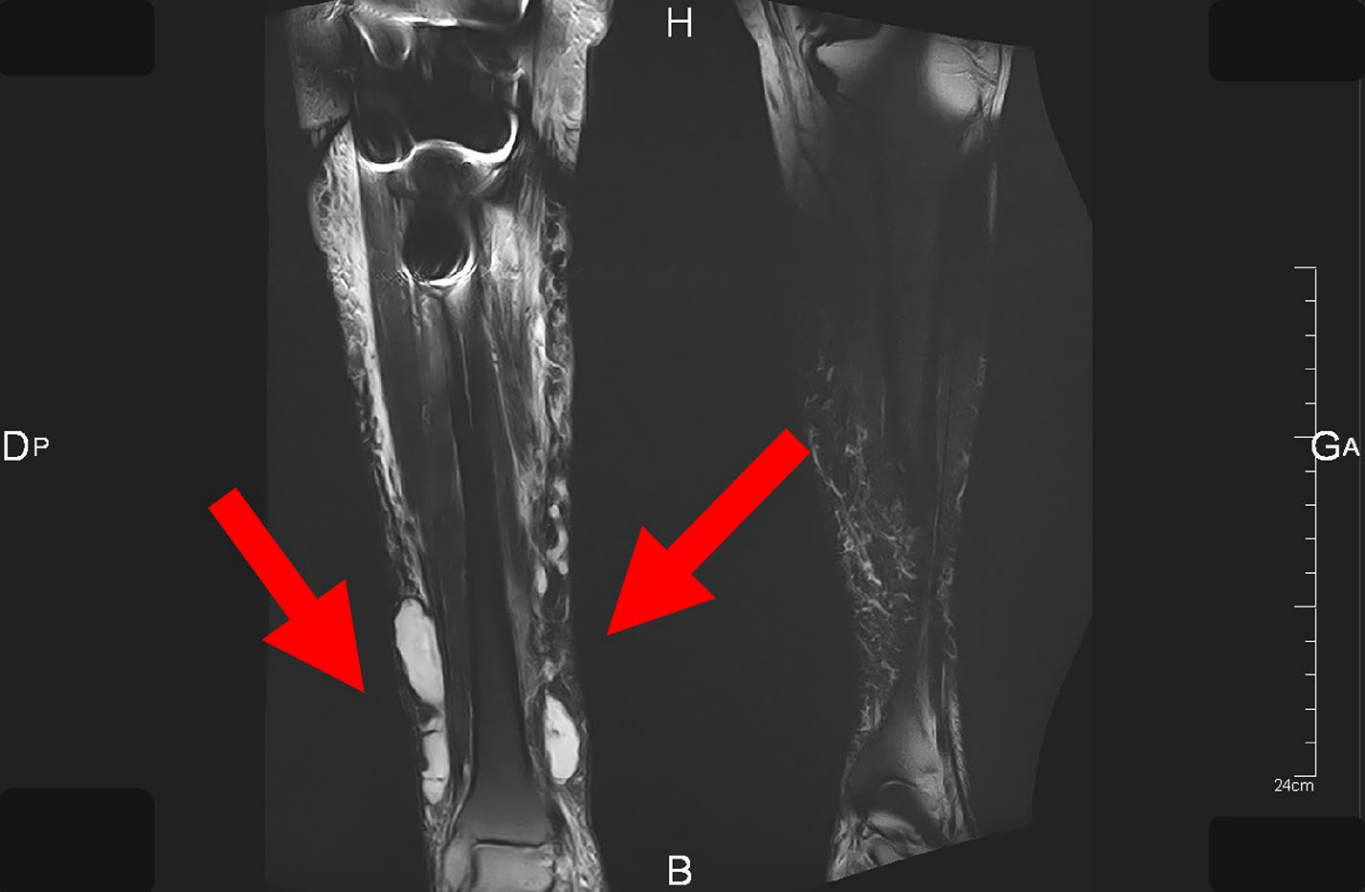
Magnetic Resonance Imaging of the legs. Coronal T2 FAT-SAT (Fat suppression) MRI sequences of the legs, showing several collections in the soft tissues of the right leg (*arrows*).

The patient rapidly developed septic shock, acute kidney injury and hyperlactatemia (7.2 mmol L^−1^, N value <1.6). Two peripheral blood cultures were positive for wild-type *P. multocida*. Isolates presented a sensibility to beta lactams, fluoroquinolones and cyclines, a resistance to lincosamides and to aminoglycosides and a decreased sensibility to macrolides. The urine sample was negative. Computed tomography (CT) and Magnetic Resonance Imaging (MRI) of the right leg (Figure 1) revealed cellulitis over the lower half of the tibia, many small fluid collections but no damage around the prosthetic knee joint. These abscesses were evacuated surgically on the day of admission, and were also positive for *P. multocida*. The piperacillin + tazobactam treatment was then replaced by ampicillin (8 g/day) and doxycycline (200 mg/day).

On day 3, the patient’s renal function had improved and norepinephrine was withdrawn.

**Figure 2:**
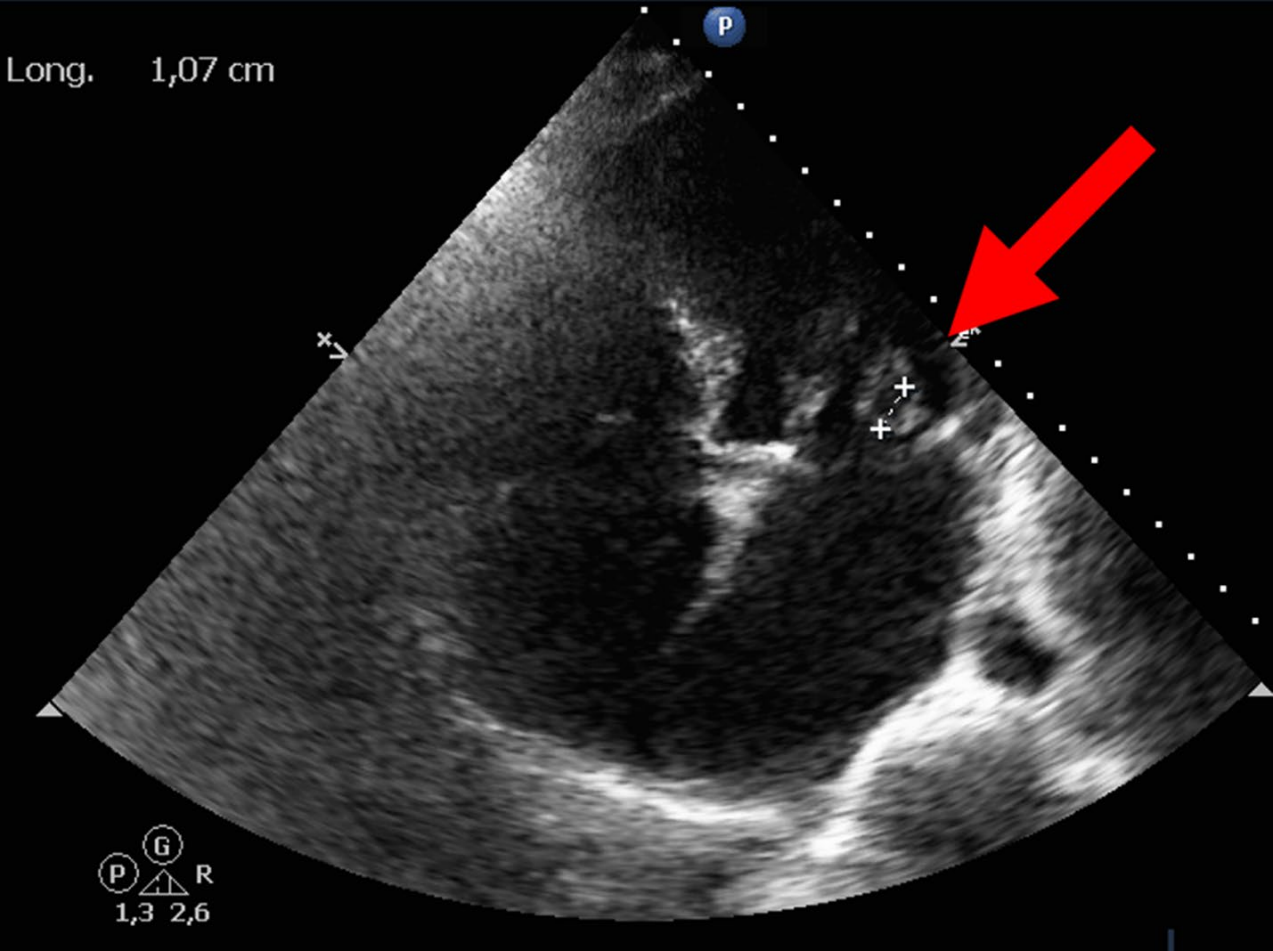
Transesophageal echocardiography. Transesophageal echocardiography, showing mobile echogenicity (*arrow*) on the small cusp of the mitral valve (prolapsing into the left ventricle).

Septic shock recurred on day four. Transthoracic echocardiography revealed intense mitral regurgitation and a left ventricular fraction ejection of 55%. Subsequent transesophageal echocardiography evidenced mobility of the small cusp of the mitral valve (Figure 2), severe mitral regurgitation, and holosystolic flow reversal in the left ventricle. Together with the patient’s blood culture results, these findings along were consistent with mitral valve endocarditis (as defined by Duke’s criteria) [2].

**Figure 3:**
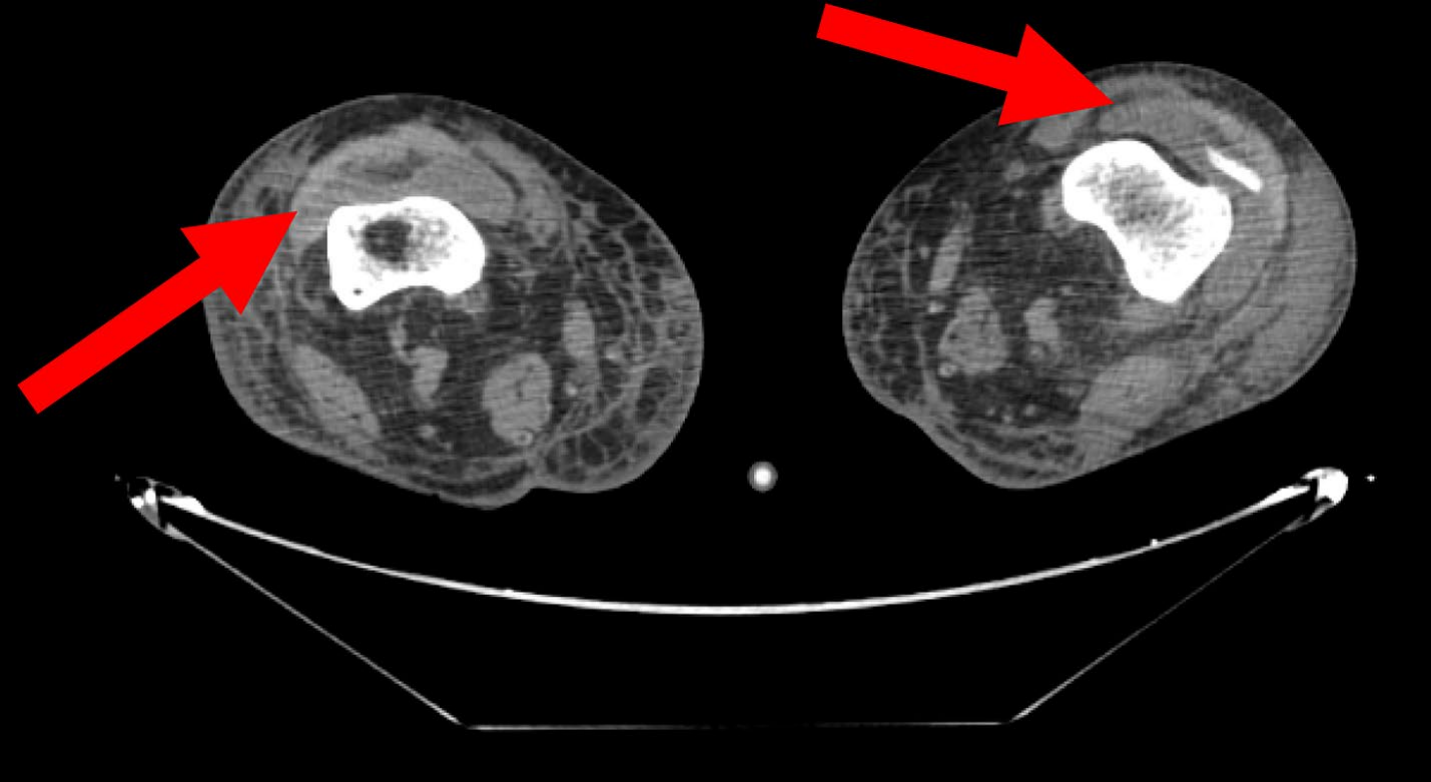
Computed tomography scan of the legs. Horizontal CT scan of the legs, showing effusions at both knee joints (*arrows*).

The patient became confused and disoriented. The results of a lumbar puncture (Cerebrospinal fluid analysis) and a brain CT scan were normal. A whole-body CT scan revealed multiple abscesses on both legs, with knee joint effusions (Figure 3). With a view to treating the infection around the prosthetic knee joint, we performed right arthrotomy and synovectomy, together with irrigation and debridement of the rest of the legs. Treatment with piperacillin + tazobactam was reintroduced and supplemented with a course of vancomycin-ciprofloxacin-amikacin.

On day 6, the patient developed treatment-refractory shock with multiple organ failure and disseminated intravascular coagulation. Despite vacuum-assisted closure, the right leg became increasing necrotic—prompting removal of the prosthetic knee joint on day 7. During surgery, the patient suffered a cardiac arrest and died despite our attempts at resuscitation.

## Discussion

There are many literature reports of *P. multocida* infections in humans. These are mainly local infections and cellulitis, although subsequent necrotizing soft tissue infections have also been reported. Oral and/or respiratory tract infections can occur in immunocompromised patients (due to diabetes mellitus, liver cirrhosis, cancer, etc.). Severe, invasive infections (such as meningitis, endocarditis, arthritis, intra-abdominal infections [3]) are less frequently described.

Although the present case did not have known liver dysfunction, he was alcoholic and presented with a dysmorphic liver on a CT scan, an elevated gamma-glutamyl transferase level and an elevated mean corpuscular volume.

Endocarditis is a rare complication of *P. multocida* sepsis [4, 5]. A review of the literature revealed a total of 34 cases, 28 of which involved native valves (13 mitral, 12 aortic, 1 pulmonary, 1 tricuspid and 1 unspecified). Only 9 cases required surgical treatment in addition to antibiotics. The outcome was fatal in 12 cases (i.e. 35% of the total). The case described here was particular in that severe bacteremia was present at several foci in cardiac, skin and joint tissue. We tried to preserve the prosthetic knee joint by combining debridement and synovectomy with intravenous antibiotics (in accordance with current guidelines) [6] but the treatment was unsuccessful. Some researchers have suggested to exchange the interspace using an antibiotic-coated component [7]. The same researchers noted that of the cases of *P. multocida*-related arthroplasty infections presented in the last decade, 62.5% had the prosthesis removed [7].

One unusual feature of the present case was the long time interval between the initial wound and hospitalization. Previous reports have described either a hematogenous infection after a scratch or bite at a remote site or reactivation of previous infection [8]. Despite surgical excision and effective antibiotic therapy, secondary endocarditis and joint infection occurred in our patient.

*Pasteurella* species are generally susceptible to several antibiotics. Penicillin is considered to be the drug of choice, although fluoroquinolones, second and third-generation cephalosporins, and carbapenem are also suitably active against *Pasteurella* [9]. In the present case, appropriate antibiotic treatment did not enable us to rush out the infection—probably because of the multiple foci and simultaneous organ damage induced by the bacteria.

## Conclusions

In conclusion, *P. multocida* infection following a poorly disinfected dog or cat bites can lead to multifocal bacteremia and a high risk of mortality. Early wound assessment (including antibiotic treatment when required) is very important in cases of animal bites, scratches or licks.

## Consent

Written informed consent was obtained from the patient’s wife for publication of this case report and accompanying images. A copy of the written consent is available for review by the Editor-in-Chief of this journal.

## Additional file

Additional file 1: CARE Checklist (2013) of information to include when writing a case report.

## Abbreviations

CT: computed tomography
MRI: magnetic resonance imaging
N value: normal value
*P. multocida*: *Pasteurella multocida*
